# Present Conditions and Prospects

**Published:** 1918-09-07

**Authors:** 


### THE ROYAL ARMY MEDICAL CORPS.

### Present Conditions and Prospects.

THE information set forth in an Educational Number of a medical newspaper is read for guidance in the choice of a career or to confirm a leaning to a particular way of life. The war has greatly modified the conditions of entrance into the R.A.M.C. and the chances of those who serve in it. The present position is transitory. It is only possible to give broad outlines of the prospects. None the less a great deal can be set forth that may help the student thinking of his future to decide whether he will choose the career the Army offers or another.

No one knows what the conditions of Army service in any branch will be after the war. Intelligent anticipation is that the pay of the officer will be raised, because it is thought that if it be not raised men will not take commissions. From a pecuniary point of view there is nothing to be gained by serving as a combatant officer, and before the war it was noticed that the entries for Sandhurst and Woolwich were scarcely equal to the vacancies. The officer's work had become hard and exacting and promotion was no longer a certainty. These conditions were keeping away many of the men of means and leisure that formerly made up a large part of the officers, and many families that had supplied officers for generations were no longer able to find money to supplement the pay of an officer. Men of brains with ambition and capital saw that the same expenditure of energy, work, and money as would lead them to wealth and honour in civil life left them

**The Royal Army Medical Corps—(*continued*).**

stranded in middle age if they joined the Army with only the half-pay of a Major. Agitation to raise the pay had commenced, and had been to a small extent successful.

It is fairly certain that the Indian Government must offer a great deal higher pay, or equivalent advantages, than it now gives its medical officers, for if it does not do so the supply of white medical officers will be greatly curtailed. Should the advantages of the I.M.S. be greater than the advantages of the R.A.M.C., there must be a levelling up of the emoluments of the latter to meet the competition.

The Government demand for medical men is an increasing demand, and so is the demand of institutions not directly under Government, and of great corporations. With this increase of demand there is no immediate probability of increase of supply. The time during which a man is not earning any money whilst preparing for qualification is long and lengthening. The capital necessary to acquire qualification is far more now than twenty years ago, and likely to be greater still; the work necessary to gain a good knowledge of modern medicine and surgery is immense, is increasing, is a strain on the strongest, and the reward of the doctor is small when set against the time, money, and work he expended in his youth. There seems, therefore, to be an increased and increasing demand and a probability of a decreasing supply. Such conditions lead to a rise of price, and therefore qualified medical men will be able, if they choose, to obtain a higher money return for their services in the future than they have received in the past in civil life or in Army service. All these considerations make it probable that the officers of the R.A.M.C. will in future receive a higher rate of pay than they do at present, and even now, though the pay is not great, the advantages of the Service are many.

"What shall I get?" is a question every man is sure to ask himself before he takes an appointment. In tabular form the rates of pay are set out below.

| | Pay. £ | s. | d. | With allowances, but exclusive of Field Allowance. £ | s. | |
|---|---|---|---|---|---|---|
| Lieutenant ... ... ... | 255 | 10 | 0 | 326 | 0 | 0 |
| Captain ... ... ... ... | 282 | 17 | 6 | 372 | 1 | 9 |
| Captain, after 7 years' full-pay service ... ... ... | 310 | 5 | 0 | 399 | 9 | 3 |
| Captain, after 10 years' full-pay service ... ... ... | 383 | 5 | 0 | 472 | 9 | 3 |
| Major ... ... ... ... | 428 | 17 | 0 | 583 | 9 | 7 |
| Major, after 3 years' service as such... ... ... ... | 474 | 10 | 0 | 629 | 2 | 1 |
| Lieut.-Colonel ... ... ... | 547 | 10 | 0 | 711 | 4 | 7 |
| Lieut.-Colonel, after 3 years' service as such ... ... | 638 | 15 | 0 | 802 | 9 | 7 |
| Colonel ... ... ... ... | 821 | 5 | 0 | 1,008 | 16 | 1 |
| Surgeon-General ... ... | 1,095 | 0 | 0 | 1,447 | 16 | 3 |

Such pay does not look very tempting. To receive £700 a year at the age of forty-five years or over and £800 a year at fifty or so does not seem a big reward for perhaps six or seven years' study of a most exacting kind and the expenditure of probably at least £1,200 in acquiring a profession.

It is not a great reward, but it must be remembered that against that can be set certainty and no professional expenses. It is nett. It is what a man has in his hand to keep himself and his wife and family. The £470 of a captain of thirty-five years is equal to a practice of £700 a year, and, beyond pay and allowances, men who work hard and are keen can almost always get some extra pay, such as £50 a year specialist's pay in the junior ranks or charge pay in the senior ranks.

But the two great pecuniary advantages are the certain income and the certain pension. After twenty years a man can retire with £365 a year, and if he stays longer he can get a better pension still, about £540 after thirty years' service and £730 to a surgeon-general of sixty years of age. The money equivalent of such annuities is about £4,500. Besides the officers' own pensions there are pensions for wives and children, all of which advantages can be expressed in cash. The Pay Warrant gives the full particulars. When all these facts are considered a man is not far wrong if he concludes that he will be doing as well in the Army as he would have done in civil life if he had bought a practice of £500 a year, worked it up to £2,000, sold it for two years' purchase at sixty, and retired on an annuity bought with the proceeds and a few hundreds of savings. A nearer comparison of the pecuniary position of the civilian and the Army doctor is scarcely possible. The comparison will not appeal to the ambitious of a fortune; but it will to many who wish to live life.

When from his retirement a man looks back on his career, and compares it with the lot of those who were young with him, unless he be in poverty, he does not think only of the money he might have made had he done as this or that friend of his youth, but more of the time he has passed, of what he has seen, of what he has done, and of the lights and shades of the long vista back down which he looks. The Army doctor, if he has used his time wisely, should look back with pleasure and satisfaction. He will recall pleasant companions, sterling comrades, jolly days when he lived in this mess or that, scenes of travel, days of sport, and to him will return memories of India, of the glamour of the East, and of a thousand scenes of here and there upon the surface of the globe. Nor will he want for memories of strenuous times, of successful operations, of good work done, of grateful patients, of epidemics, of cholera camps, of fighting, wounds and death. There must be many men in the neighbourhood of Cavendish Square near to the summit of the ambition with which they left the examination halls who, when they talk with men of their year who have thrown in their lot with the Army, wonder if, after all, despite his smaller cash takings and his comparative obscurity, the old friend who has lived so wandering a life has not really had the best of it. Still more must this be so of him who has made a fair practice in some great factory town or tourist-ridden watering-place. Is the constant rush and round from this patient to that through the same dull familiar streets and the break of a fortnight's yearly holiday as satisfactory to look back on as another life that might have been? Who knows! We all have our regrets and our compensations.

